# Genome sequences of key bacterial symbionts of entomopathogenic nematodes: *Xenorhabdus cabanillasii* DSM17905, *Xenorhabdus ehlersii* DSM16337, *Xenorhabdus japonica* DSM16522, *Xenorhabdus koppenhoeferii* DSM18168, and *Xenorhabdus mauleonii* DSM17908

**DOI:** 10.1128/MRA.00548-23

**Published:** 2023-09-15

**Authors:** Raegan Robertson, Katie Conrad, Baarik Ahuja, Markus Göker, Richard L. Hahnke, Alex Spunde, Natalia N. Ivanova, Rekha Seshadri, Craig Stephens

**Affiliations:** 1 Santa Clara University, Santa Clara, California, USA; 2 Leibniz Institute DSMZ, German Collection of Microorganisms and Cell Cultures, Braunschweig, Germany; 3 DOE Joint Genome Institute, Lawrence Berkeley National Laboratory, Berkeley, California, USA; 4 Department of Biology, Santa Clara University, Santa Clara, California, USA; 5 Department of Public Health, Santa Clara University, Santa Clara, California, USA; University of Maryland School of Medicine, Baltimore, Maryland, USA

**Keywords:** Xenorhabdus, secondary metabolism, entomopathogen, nematodes

## Abstract

*Xenorhabdus* species are bacterial symbionts of entomopathogenic *Steinernema* nematodes, in which they produce diverse secondary metabolites implicated in pathogenesis. To expand resources for natural product prospecting and exploration of host-symbiont-pathogen relationships, the genomes of *Xenorhabdus cabanillasi, Xenorhabdus ehlersii*, *Xenorhabdus japonica*, *Xenorhabdus koppenhoeferii*, and *Xenorhabdus mauleonii* were analyzed.

## ANNOUNCEMENT

Species of the genus *Xenorhabdus* (Bacteria, Pseudomonadota, Gammaproteobacteria, Enterobacterales, and Morganellaceae) (1) are Gram-negative bacteria that form symbiotic relationships with entomopathogenic nematodes of the genus *Steinernema* (2). During the *Xenorhabdus–Steinernema* life cycle, insect larvae are infected and killed, including species with significant ecological and economic impacts, such that some *Steinernema* have been employed as biocontrol agents (2). The pathogenicity of the nematode host is dependent on secondary metabolite production by the *Xenorhabdus* symbiont (3), making these bacteria a target for prospecting for novel bioactive compounds. With 27 validly published species to date (1), there is great genetic diversity in the genus *Xenorhabdus* (4), and the genomes described here expand resources for bioprospecting and exploration of complex symbiont-host-pathogen relationships.

*Xenorhabdus cabanillasii* DSM17905 was originally isolated from the nematode *Steinernema riobrave* in the US (Texas), *Xenorhabdus ehlersii* DSM16337 was isolated from *Steinernema serratum* in China, *Xenorhabdus japonica* DSM16522 from *Steinernema serratum kushidai* in Japan, *Xenorhabdus koppenhoeferii* DSM18168 from *Steinernema serratum scarabaei* in the US (New Jersey), and *Xenorhabdus mauleonii* DSM17908 from an unidentified *Steinernema* species in St. Vincent (Caribbean) (5). All isolates were supplied by the Leibniz Institute DSMZ (6). Cultures were grown aerobically at 28°C in DSMZ medium 1 (https://mediadive.dsmz.de/) (7), with the exception of DSM16522, which was grown in DSMZ medium 535. Genomic DNA from *X. ehlersii* was isolated using MasterPure Gram Positive DNA Purification Kit (Epicentre MGP04100). DNA from other species was isolated using Jetflex Genomic DNA Purification Kit (GENOMED 600100). DNA is available from DSMZ through the DNA Bank Network (7).

Genomes of *X. cabanillasii and X. ehlersii* were analyzed at JGI using Pacific Biosciences (PacBio) sequencing technology (8). DNA (2 ug) was treated to remove single-stranded ends and repair damage, followed by A-tailing and ligation with PacBio adapters using SMRTbell Template Prep Kit 1.0. Final size selection for 6–10 kb templates used the Sage BluePippin system. PacBio Sequencing primer was annealed to the SMRTbell template library, and Version P6 sequencing polymerase was bound to them. Libraries were sequenced on a PacBio RSII sequencer using Version-C4 chemistry and 1 × 120 sequencing movie run times. Reads were assembled using HGAP (smrtanalysis/2.3.0 p5, HGAP3) (9). Read N_50_ was 5,289 bp for *X. cabanillasii* and 5,660 bp for *X. ehlersii*.

For *X. japonica*, *X. koppenhoeferii*, and *X. mauleonii*, Illumina (10) 300 bp insert shotgun libraries were constructed from 100 ng of DNA that had been sheared using the Covaris LE220, and size was selected using SPRI beads (Beckman Coulter). Library construction used the KAPA Library Preparation Kit (KAPA Biosystems) for the Illumina platform, which includes end-repair, A-tailing, and ligation of Illumina compatible adapters (IDT, Inc) as recommended by the manufacturer. Libraries were quantified using KAPA Biosystem’s Library Quantification Kit for Illumina platforms (Roche) and run on a Roche LightCycler480 real-time PCR instrument. Quantified libraries were multiplexed and prepared for sequencing on the Illumina HiSeq 2500 platform utilizing a TruSeq paired-end cluster kit, v4, and Illumina’s cBot instrument to generate a clustered flow cell for sequencing. Sequencing was performed using HiSeq TruSeq SBS sequencing kits, v4, following a 2 × 150 indexed run recipe. Raw reads were filtered using BBDuk (11). Reads with more than one “N,” quality scores averaging <8 (before trimming), or lengths <51 bases (after trimming) were discarded. The remaining reads were mapped to masked versions of human, cat, and dog references using BBMAP and discarded if identity exceeded 95%. Filtered Illumina reads were assembled using SPAdes (version 3.6.2) (12). Parameters for the SPAdes assembly were —cov–cutoff auto —phred–offset 33 –t 8 –m 40 —careful –k 25,55,95 —12. Assembled contigs <1 kb were discarded. Using CheckM2 (13), all genomes were 100% complete. The final assemblies were annotated by the JGI genome annotation pipeline (14).

Final genome statistics and links to NCBI genome and sequencing data archives for the five *Xenorhabdus* species are summarized in Table 1. Data and detailed reports can also be downloaded from the JGI Genome portal and the JGI Integrated Microbial Genomes with Microbiomes (IMG/M) system (15).

**TABLE 1:** *Xenorhabdus* genome assembly statistics and accessions

	*X. cabanillasii* DSM17905	*X. ehlersii* DSM16337	*X. japonica* DSM16522	*X. koppenhoeferii* DSM18168	*X. mauleonii* DSM17908
Sequencing platform	Pacific Biosciences RS2	Pacific Biosciences RS2	Illumina HiSeq 2500	Illumina HiSeq 2500	Illumina HiSeq 2500
# reads used in assembly	148,449	130,742	10,000,000	10,000,000	9,878,486
Coverage	87.6 ×	90.3 ×	428 ×	477 ×	349 ×
# assembled scaffolds(>1 kb)	1	9	92	92	95
Total scaffold sequence length	4,335,622 bp	4,058,264 bp	3,561,198 bp(0.01% gaps)	3,182,127 bp(0.02% gaps)	5,119,810 bp (0.04% gaps)
Contig N50	4,335,622 bp	941,920 bp	80,906 bp	70,143 bp	178,279 bp
Largest Contig	4,335,622 bp	1,272,290 bp	220,581 bp	188,443 bp	440,959 bp
GC content (%)	42.9	43.8	42.7	43.0	43.9
# predicted CDS	3,754	3,701	3,228	2,794	4,393
GenBank accession number	NZ_RAQI00000000.1	NZ_QTUB00000000.1	NZ_FOVO00000000.1	NZ_FPBJ00000000.1	NZ_NITY00000000.1
NCBI SRA accession number	SRX3886576	SRX3785650	SRX2156714	SRX2156721	SRX2156707
JGI IMG/G taxon ID	2778260932	2772190835	2684622846	2684622845	2684622849

AntiSMASH 7.0 was used to survey genomes for biosynthetic gene clusters (BGCs) potentially involved in the synthesis of bioactive secondary metabolites (16). At least one non-ribosomal peptidyl synthase (NRPS) BGC most similar to that involved in synthesis of xenoamicins A and B (17) was conserved in all five genomes. Also common were BGCs encoding, for example, NRPS-like systems for assembly of the antibiotics nematophin (18) and safracin (19), a Type II polyketide synthase for the synthesis of aryl polyenes (20), and siderophores of the putrebactin/avaroferrin class (21). Each genome also contained unique BGCs whose products are yet unknown.
